# Supplementary material for: Analysis of PPARγ Signaling Activity in Psoriasis
Source: Int J Mol Sci. 2021 Aug 10;22(16):8603. doi: 10.3390/ijms22168603 (PMC8395241; doi:10.3390/ijms22168603)
Supplement: Supplementary file 1 [file ijms-22-08603-s001.zip › Supplemental materials_Analysis of PPARg signaling activity in psoriasis/Pathway models/Models images and html files/Differentiation of psoriatic T cells/7181.html]

SMAD3


# Protein SMAD3

|  |  |
| --- | --- |
| URN | urn:agi-llid:4088 |
| Total Entities | 0 |
| Connectivity | 4919 |
| Name | SMAD3 |
| Description | SMAD family member 3 |
| Notes | The protein encoded by this gene belongs to the SMAD, a family of proteins similar to the gene products of the Drosophila gene 'mothers against decapentaplegic' (Mad) and the C. elegans gene Sma. SMAD proteins are signal transducers and transcriptional modulators that mediate multiple signaling pathways. This protein functions as a transcriptional modulator activated by transforming growth factor-beta and is thought to play a role in the regulation of carcinogenesis. [provided by RefSeq, Apr 2009] |
| Primary Cell Localization | Nucleus |
| Class | Transcription factor |
| ObjectType | Protein |

---

|  |  |
| --- | --- |
| Pathway | Osteoarthritis |
|  | Cortisol in Resolving Inflammation |
|  | Immunoglobulin Class-Switch Recombination Activation |
|  | Treg-Cell Differentiation |
|  | AHR Signaling in Treg and Dendritic Cells Function |
|  | AHR Signaling in Th17 Cells Function |
|  | Th17-Cell Differentiation |
|  | Vascular Endothelial Cell Activation by Growth Factors |
|  | Vascular Smooth Muscle Cell/Pericyte Differentiation and Proliferation |
|  | PRL Expression Regulation by BMP4 and TGFB1 |
|  | Activin, Inhibin and Follistatin Action on Pituitary Gland |
|  | G1/S Damage Checkpoint |
|  | Cell Cycle Overiew |
|  | Cardiomyocyte Dysfunction |
|  | Dilated Cardiomyopathy |
|  | Cardiomyocyte Hypertrophy |
|  | Hypertrophic Cardiomyopathy |
|  | Proteins Involved in Arterial Hypertension |
|  | Proteins Involved in Pulmonary Hypertension |
|  | BMP/TGF-beta Signaling Impairment in Pulmonary Hypertension |
|  | BMP2 and Non-Canonical WNT Signaling in Pulmonary Artery Endothelial Cells |
|  | Endothelial Cell Dysfunction in Pulmonary Hypertension |
|  | Lymphocyte Mediated Myocardial Injury in Myocarditis |
|  | Androgens in Sebocyte Maturation |
|  | Androgens Promote Scalp Dermal Papilla Regression |
|  | T-Cells Differentiation Block in Psoriasis |
|  | TGF-beta Signaling in Skin Aging |
|  | Treg-Cell Function in Diabetes Mellitus Type 1 |
|  | non-Suppressive Treg-Cell in Diabetes Mellitus Type 1 |
|  | TLR2 Signaling in Treg-Cell in Type 1 Diabetes (Animal Model) |
|  | Treg-Cell Activation in Diabetes Mellitus |
|  | Tubular Cell Dysfunction in Progressive Diabetic Nephropathy |
|  | Endothelial Cell Dysfunction in Progressive Diabetic Nephropathy |
|  | Proteins Involved in Diabetic Nephropathy |
|  | Mesangial Cell Dysfunction in Diabetic Nephropathy |
|  | NKX2-1 in Thyroid Dysgenesis (Hypothesis) |
|  | GLIS3 Targets in Thyroid Dysgenesis (Hypothesis) |
|  | PAX8 Targets in Thyroid Dysgenesis (Hypothesis) |
|  | Proteins Involved in Obesity |
|  | Prolactinoma Overiew |
|  | Metastatic Colorectal Cancer |
|  | GUCY2C Signaling in Colorectal Cancer |
|  | Peutz-Jeghers Syndrome |
|  | Chronic Myeloid Leukemia |
|  | Vascularization in Hepatocellular Carcinoma |
|  | NOTCH Signaling in Hepatocellular Carcinoma |
|  | Hepatocellular Carcinoma |
|  | Growth Factor Signaling in Hepatocellular Carcinoma |
|  | Pancreatic Ductal Carcinoma |
|  | Pancreatic Neuroendocrine Tumors |
|  | STK11 Signaling in Pancreatic Neoplasms |
|  | TGFBR Signaling in Pancreatic Neoplasms |
|  | Th17-Cell Differentiation in Asthma |
|  | Osteoblast Function Decline in Gout |
|  | Proteins Involved in Osteoarthritis |
|  | TGFB2 Signaling Impairement in Osteoarthritis |
|  | Th17-Cell and Th1 Immune Responsein Psoriatic Arthritis |
|  | Synovial Fibroblast Proliferation in Rheumatoid Arthritis |
|  | Autocrine Cytokine/Chemokine Loops in Systemic Scleroderma |
|  | Skin Fibrosis |
|  | Th17-Cell Numbers Reduction in HIV |
|  | Hereditary Hemorrhagic Telangiectasia |
|  | Myostatin in Skeletal Muscles in Muscular Dystrophies |
|  | Myostatin-IGF1 Crosstalk in Skeletal Muscles in Muscular Dystrophies |
|  | Genes with Mutations Associated with Osteoarthritis |
|  | Bone Loss in Osteoporosis |
|  | Hyperparathyroidism, Secondary Effect |
|  | Glioma Stem Cell Program Activation |
|  | Medulloblastoma |
|  | Proteins Involved in Breast Cancer Related to ESR1 Signaling Pathway |
|  | Proteins Involved in Breast Cancer Related to NOTCH Signaling Pathway |
|  | Proteins Involved in Breast Cancer Related to ERBB2/VEGFR/Akt Signaling Pathway |
|  | Proteins Involved in Breast Cancer Related to IGF1R/Akt Signaling Pathway |
|  | Breast Cancer |
|  | NOTCH1 Signaling in Breast Cancer |
|  | Proteins Involved in Female Infertility |
|  | Ovarian Cancer |
|  | INHA Gene Mutations in Granulosa Cell Proliferation |
|  | FSH Hyperproduction Due to INHA Mutation in Primary Ovarian Insufficiency |
|  | BMP Signaling Impairment in Granulosa Cell in POF |
|  | Prostate Cancer |
|  | Cytokines Trigger Otitis Media |
|  | Proteins Involved in Pulmonary Emphysema |
|  | Podocyte Dysfunction in Glomerulonephritis |
|  | Mesangial Cell Dysfunction in Glomerulonephritis |
|  | Interstitial Fibroblasts in Pyelonephritis |
|  | BMP6/ACVR2A Expression Targets |
|  | BMP7/ACVR2 Expression Targets |
|  | Elevated Receptors -> Expression Targets in Bone |
|  | Dexamethasone Induced Osteoporosis |
|  | EGF -> CTNN Expression Targets |
|  | EGF -> AP-1/ATF Expression Targets |
|  | EGF -> STAT Expression Targets |
|  | FGF2 -> STAT Expression Targets |
|  | FGF2 -> AP-1/CREB/CREBBP/ELK/SRF/MYC Expression Targets |
|  | Proteins with Altered Expression in Cancer-Associated Sustaining of Proliferative Signaling |
|  | Genes with Mutations in Cancer-Associated Sustaining of Proliferative Signaling |
|  | DNA Damage Checkpoint Impairment in Cancer |
|  | IL23A and IL17A Provoke Cancer-Associated Inflammation |
|  | N2 Neutrophils in Tumor-Promoting Inflammation and Tumor Progression |
|  | TGFB Signaling activation by Blocking of Tumor Suppressors |
|  | Genes with Mutations in Cancer-Associated Evading of Growth Suppressors |
|  | Proteins with Altered Expression in Cancer-Associated Evading of Growth Suppressors |
|  | HIF1A in Vasculogenic Mimicry of Cancer |
|  | FBLN5 Potentiates Migration and Proliferation of Endothelial Cells in Cancer |
|  | TGFB Family in Epithelial to Mesenchymal Transition in Cancer |
|  | Skeletal Muscle Wasting in Cancer Cachexia |
|  | Treg Cells Promote Immunosuppression in Cancer Immune Escape |
|  | INHBB/ACVR2 Expression Target |
|  | INHBA/ACVR2/ACVR1 Expression Targets |
|  | NOTCH -> Expression Targets in Skin |
|  | NOTCH -> Expression Targets in Thymus |
|  | NGF -> SMAD3/NF-kB Expression Targets |
|  | BDNF Expression Targets |
|  | NTF3 Expression Targets |
|  | NTF4 Expression Targets |
|  | NOTCH -> SMAD3 Signaling |
|  | ADAM17 Expression Targets |
|  | NOTCH Expression Targets |
|  | NTRK -> AP-1/CREB/ELK/SRF/MYC/SMAD3/TP53 Signaling |
|  | TGFBR/BMPR -> SMAD2/3 Signaling |
|  | ActivinR -> SMAD2/3 Signaling |
|  | TGFBR -> MEF/MYOD/MYOG Signaling |
|  | TGFBR1/AP-1 Signaling |
|  | MSTN/ACVR2/ACVR1 Expression Targets |
|  | NODAL/ACVR2B Expression Targets |
|  | NOTCH Receptors -> Receptors Expression Targets in Lung |
|  | JNK/MAPK Signaling |
|  | TGF-beta Signaling |
|  | TGFB2-TGFBR1 Expression Targets |
|  | TGFB3-TGFBR1 Expression Targets |
|  | TGFB1-TGFBR1 Expression Targets |
|  | TGFB1-TGFBR1/AP-1 Expression Targets |
|  | TGFB1-ACVRL1 Expression Targets |
|  | ROS in Angiotensin Mediated Cardiovascular Remodeling and Hypertrophy |
|  | n |
|  | New Pathway (2) |
|  | PPAR Psoriasis |
|  | prarg negative regulators, ps-positive |
|  | pprarg neg, uknown targets, ps-positive |
|  | prarg neg,ukn expres targets, ps-positive |
|  | PPARG negative regulators |
|  | PPARG negative targets |
|  | pparg expr target\_regulators |
|  | Neighbors of disease exacerbation |
|  | Neighbors of tumor progression |
|  | Neighbors of cancer progression |
|  | Neighbors of overall survival |
|  | Neighbors of disease severity |
|  | Neighbors of mortality |
|  | Neighbors of survival rate |
|  | Neighbors of infarct size |
|  | Neighbors of survival time |
|  | Neighbors of insulin sensitivity |
|  | Neighbors of clinical stage |
|  | Neighbors of lethality |
|  | Neighbors of recurrence-free survival |
|  | Neighbors of disease-free survival |
|  | Neighbors of tumor response |
|  | Neighbors of macrophage count |
|  | Neighbors of bone mass |
|  | Neighbors of body weight |
|  | Neighbors of body weight gain |
|  | Neighbors of cardiovascular risk |
|  | Neighbors of microvessel density |
|  | Neighbors of tumor promotion |
|  | Neighbors of neutrophil count |
|  | Neighbors of glucose tolerance |
|  | Neighbors of fibrosis progression |
|  | Neighbors of mortality rate |
|  | Neighbors of bone mineral density |
|  | Neighbors of adverse outcome |
|  | Neighbors of cell density |
|  | Neighbors of apoptosis rate |
|  | Neighbors of blood pressure |
|  | Neighbors of vascular reactivity |
|  | Neighbors of muscle mass |
|  | Neighbors of cancer risk |
|  | Neighbors of systolic blood pressure |
|  | Neighbors of tumor resistance |
|  | Neighbors of blood glucose |
|  | Neighbors of renal hypertrophy |
|  | Neighbors of energy expenditure |
|  | Neighbors of radiosensitivity |
|  | Neighbors of left ventricular ejection fraction |
|  | Neighbors of fertility |
|  | Neighbors of lymphocyte count |
|  | Neighbors of insulin level |
|  | Neighbors of fibroblast phenotype |
|  | Neighbors of disease exacerbation |
|  | Neighbors of tumor progression |
|  | Neighbors of cancer progression |
|  | Neighbors of overall survival |
|  | Neighbors of disease severity |
|  | Neighbors of mortality |
|  | Neighbors of survival rate |
|  | Neighbors of infarct size |
|  | Neighbors of survival time |
|  | Neighbors of insulin sensitivity |
|  | Neighbors of clinical stage |
|  | Neighbors of lethality |
|  | Neighbors of recurrence-free survival |
|  | Neighbors of disease-free survival |
|  | Neighbors of tumor response |
|  | Neighbors of macrophage count |
|  | Neighbors of bone mass |
|  | Neighbors of body weight |
|  | Neighbors of body weight gain |
|  | Neighbors of cardiovascular risk |
|  | Neighbors of microvessel density |
|  | Neighbors of tumor promotion |
|  | Neighbors of neutrophil count |
|  | Neighbors of glucose tolerance |
|  | Neighbors of fibrosis progression |
|  | Neighbors of mortality rate |
|  | Neighbors of bone mineral density |
|  | Neighbors of adverse outcome |
|  | Neighbors of cell density |
|  | Neighbors of apoptosis rate |
|  | Neighbors of blood pressure |
|  | Neighbors of vascular reactivity |
|  | Neighbors of muscle mass |
|  | Neighbors of cancer risk |
|  | Neighbors of systolic blood pressure |
|  | Neighbors of tumor resistance |
|  | Neighbors of blood glucose |
|  | Neighbors of renal hypertrophy |
|  | Neighbors of energy expenditure |
|  | Neighbors of radiosensitivity |
|  | Neighbors of left ventricular ejection fraction |
|  | Neighbors of fertility |
|  | Neighbors of lymphocyte count |
|  | Neighbors of insulin level |
|  | Neighbors of fibroblast phenotype |
|  | Neighbors of cell development |
|  | Neighbors of immune response |
|  | Neighbors of cell population |
|  | Neighbors of T-cell development |
|  | Neighbors of inflammatory response |
|  | Neighbors of wound healing |
|  | Neighbors of angiogenesis |
|  | Neighbors of innate immune response |
|  | Neighbors of cell function |
|  | Neighbors of macrophage activation |
|  | Neighbors of cell formation |
|  | Neighbors of epithelial to mesenchymal transition |
|  | Neighbors of immunity |
|  | Neighbors of cell proliferative response |
|  | Neighbors of chemotaxis |
|  | Neighbors of cell survival |
|  | Neighbors of osteoclast differentiation |
|  | Neighbors of cell phenotype |
|  | Neighbors of T-cell proliferation |
|  | Neighbors of tissue remodeling |
|  | Neighbors of cell adhesion |
|  | Neighbors of T-cell activation |
|  | Neighbors of endothelial cell proliferation |
|  | Neighbors of fibrogenesis |
|  | Neighbors of tumor growth |
|  | Neighbors of tissue repair |
|  | Neighbors of cell count |
|  | Neighbors of cell infiltration |
|  | Neighbors of SMC proliferation |
|  | Neighbors of lumen formation |
|  | Neighbors of endothelial cell migration |
|  | Neighbors of adipogenesis |
|  | Neighbors of lipid storage |
|  | Neighbors of cell damage |
|  | Neighbors of aging |
|  | Neighbors of cell motility |
|  | Neighbors of monocyte migration |
|  | Neighbors of fibroblast proliferation |
|  | Neighbors of adipocyte differentiation |
|  | Neighbors of microglial activation |
|  | Neighbors of regeneration |
|  | Neighbors of cell migration |
|  | Neighbors of cancer growth |
|  | Neighbors of smooth muscle cell migration |
|  | Neighbors of transendothelial migration |
|  | Neighbors of phagocytosis |
|  | Neighbors of leukocyte migration |
|  | Neighbors of osteoclast formation |
|  | Neighbors of cell invasion |
|  | Neighbors of heart function |
|  | Neighbors of hemopoiesis |
|  | Neighbors of ossification |
|  | Neighbors of T-cell function |
|  | Neighbors of immune system function |
|  | Neighbors of lung development |
|  | Neighbors of autophagy |
|  | Neighbors of transcription activation |
|  | Neighbors of senescence |
|  | Neighbors of tumor immunity |
|  | Neighbors of hepatocyte proliferation |
|  | Neighbors of cell transdifferentiation |
|  | Neighbors of neuroprotection |
|  | Neighbors of stem cell differentiation |
|  | Neighbors of neuronal death |
|  | Neighbors of cell growth |
|  | Neighbors of ROS generation |
|  | Neighbors of epithelial cell proliferation |
|  | Neighbors of cancer cell growth |
|  | Neighbors of hemato-encephalic barrier |
|  | Neighbors of cellular senescence |
|  | Neighbors of colony formation |
|  | Neighbors of cell differentiation |
|  | Neighbors of keratinocyte proliferation |
|  | Neighbors of cell homeostasis |
|  | Neighbors of psoriasis |
|  | Neighbors of inflammatory disease |
|  | Neighbors of atherosclerosis |
|  | Neighbors of fibrosis |
|  | Neighbors of colitis |
|  | Neighbors of injury |
|  | Neighbors of vascular remodeling |
|  | Neighbors of metastasis |
|  | Neighbors of carcinogenesis |
|  | Neighbors of chronic inflammation |
|  | Neighbors of inflammation |
|  | Neighbors of tumor microenvironment |
|  | Neighbors of pathological angiogenesis |
|  | Neighbors of obesity |
|  | Neighbors of vascular disease |
|  | Neighbors of diabetes mellitus |
|  | Neighbors of pneumonia |
|  | Neighbors of osteoarthritis |
|  | Neighbors of liver fibrosis |
|  | Neighbors of myocardial infarction |
|  | Neighbors of reperfusion injury |
|  | Neighbors of airway inflammation |
|  | Neighbors of insulin resistance |
|  | Neighbors of infection |
|  | Neighbors of vascular injury |
|  | Neighbors of renal injury |
|  | Neighbors of macrophage infiltration |
|  | Neighbors of liver injury |
|  | Neighbors of sepsis |
|  | Neighbors of death |
|  | Neighbors of autoimmune disease |
|  | Neighbors of ischemic stroke |
|  | Neighbors of dermatitis |
|  | Neighbors of neutrophil infiltration |
|  | Neighbors of heart disease |
|  | Neighbors of diabetic nephropathy |
|  | Neighbors of lung injury |
|  | Neighbors of systemic sclerosis |
|  | Neighbors of hepatocellular carcinoma |
|  | Neighbors of autoimmunity |
|  | Neighbors of gastric cancer |
|  | Neighbors of colorectal cancer |
|  | Neighbors of cardiac remodeling |
|  | Neighbors of breast cancer |
|  | Neighbors of systemic lupus erythematosus |
|  | Neighbors of melanoma |
|  | Neighbors of collagen-induced arthritis |
|  | Neighbors of cartilage degeneration |
|  | Neighbors of interstitial fibrosis |
|  | Neighbors of neoplasm invasion |
|  | Neighbors of kidney disease |
|  | Neighbors of heart failure |
|  | Neighbors of lung disease |
|  | Neighbors of pulmonary fibrosis |
|  | Neighbors of cardiovascular disease |
|  | Neighbors of cancer |
|  | Neighbors of type 2 diabetes |
|  | Neighbors of neoplasm |
|  | Neighbors of neointima |
|  | Neighbors of vasculitis |
|  | Neighbors of atrophy |
|  | Neighbors of diet-induced obesity |
|  | Neighbors of myocardial fibrosis |
|  | Neighbors of coronary artery disease |
|  | Neighbors of rupture |
|  | Neighbors of prostate cancer |
|  | Neighbors of hypertrophy |
|  | Neighbors of osteolysis |
|  | Neighbors of ovarian cancer |
|  | Neighbors of hypertension |
|  | Neighbors of intimal hyperplasia |
|  | Neighbors of proteinuria |
|  | Neighbors of hepatic stellate cell |
|  | Neighbors of lung |
|  | Neighbors of liver |
|  | Neighbors of airway |
|  | Neighbors of aorta |
|  | Neighbors of esophagus |
|  | Neighbors of ovary |
|  | Neighbors of immune system |
|  | Neighbors of glands |
|  | Neighbors of ankle joint |
|  | Neighbors of knee joint |
|  | Neighbors of hip region |
|  | Neighbors of NF-kB family |
|  | Neighbors of oxidized LDL |
|  | Neighbors of PPAR |
|  | Neighbors of Jun/Fos |
|  | Neighbors of PKC |
|  | Neighbors of mitogen-activated protein kinase |
|  | Neighbors of transforming growth factor |
|  | Neighbors of Ras GTPase |
|  | Neighbors of PKA |
|  | Neighbors of ovalbumin |
|  | Neighbors of LDL |
|  | Neighbors of NF-AT family |
|  | Neighbors of NAD(P)H oxidase |
|  | Neighbors of SMAD subfamily |
|  | Neighbors of LXR |
|  | Neighbors of estrogen receptor |
|  | Neighbors of PDGF-BB |
|  | Neighbors of Notch |
|  | Neighbors of heat shock protein 90 |
|  | Neighbors of Ang II receptor |
|  | Neighbors of proteasome endopeptidase complex |
|  | Neighbors of class nuclear receptor with C4 zinc fingers |
|  | Neighbors of non-coding RNA |
|  | Neighbors of histone H3 |
|  | Neighbors of retinoid-X receptor subfamily |
|  | Neighbors of RA receptor |
|  | comon ps\_pos, pprarg\_neg targets |
|  | Model of PPARG signaling in psoriasis |
|  | PPARG negative regulators and targets |
|  | Model of PPARG related pathways in psoriasis (short version) |
|  | sars-cov binding |
|  | sars-cov no binding |
|  | New Pathway |
|  | 1\_Differentiation of psoriatic T cells |
|  | 3\_TGFB signaling provokes endochondral ossification with osteophyte formation |
|  | 3\_The role of interstitial fibroblasts in pyelonephritis |
|  | 3\_Mesangial cell dysfunction in glomerulonephritis |
|  | 2\_Podocyte dysfunction in glomerulonephritis |
|  | 1\_Disruption of luteinizing hormone and follicle-stimulating hormone secretion in PCOS |
|  | 3\_Increased proliferation of pulmonary artery smooth muscle cells (PASMCs) in familial forms of pulmonary hypertension |
|  | 2\_Cardiomyocyte hypertrophy |
|  | 3\_Cardiomyocyte dysfunction unrelated to cell junctions in ARVD |
|  | 2\_Defective tolerance of autoreactive T-cell in T1D |
|  | DIPG non histone mutations |
|  | DIPG overview\_2 |
|  | Differentiation of psoriatic T cells |

---

|  |  |
| --- | --- |
| Group | Genes with Mutations Associated with Inflammatory Bowel Diseases |
|  | Genes with Mutations in GWA Studies of Asthma |
|  | binding partnetrs for SARSs |
|  | other partners for SARS |

---

|  |  |
| --- | --- |
| MedScan ID | 4088 |

---

|  |  |
| --- | --- |
| LocusLink ID | 4088 |
|  | 17127 |
|  | 25631 |

---

|  |  |
| --- | --- |
| Alias | hMAD-3 |
|  | HSPC193 |
|  | OTTMUSP00000023918 |
|  | DKFZP586N0721 |
|  | MAD (mothers against decapentaplegic, Drosophila) homolog III |
|  | MAD homolog 3 |
|  | sma- and mad-related protein 3 |
|  | HsT17436 |
|  | transcription factor Smad3 |
|  | Smad 3 |
|  | OTTMUSP00000023917 |
|  | MAD (mothers against decapentaplegic, Drosophila) homolog 3 |
|  | JV15 II |
|  | LDS1C |
|  | MAD (mothers against decapentaplegic homolog III |
|  | homolog of Drosophila Mothers against decapentaplegic III |
|  | JV15-L |
|  | Mothers against decapentaplegic, drosophila, homolog of, III |
|  | SMAD, mothers against DPP homolog 3 |
|  | JV15-2 |
|  | AU022421 |
|  | Mothers against DPP homolog III |
|  | Mothers against DPP homolog 3 |
|  | Mothers against decapentaplegic, drosophila, homolog of, 3 |
|  | JV15-Ls |
|  | homolog of Drosophila Mothers against decapentaplegic 3 |
|  | Mothers against decapentaplegic homolog 3 (SMAD 3) |
|  | MGC60396 |
|  | SMAD, mothers against DPP homolog III |
|  | Smad3 transcription factor |
|  | MAD homolog 3 (Drosophila) |
|  | DKFZp686J10186 |
|  | mothers against decapentaplegic homolog 3 |
|  | mad homolog JV15-2 |
|  | hSMAD3 |
|  | MAD homolog III |
|  | MAD, mothers against decapentaplegic homolog 3 (Drosophila) |
|  | MAD (mothers against decapentaplegic homolog 3 |
|  | RP23-63E8.1 |
|  | Madh3 |
|  | mMad3 |
|  | LDS3 |
|  | MAD, mothers against decapentaplegic homolog 3 |
|  | mad protein homolog |
|  | mad3 |
|  | SMAD family member 3 |
|  | Mothers against DPP homolog III |
|  | Mothers against DPP homolog 3 |
|  | SMAD3 |
|  | JV15-2s |
|  | hSMAD3s |
|  | mother against decapentaplegic homolog 3 |

---

|  |  |
| --- | --- |
| GO ID | 0017151 |
|  | 0001228 |
|  | 0003700 |
|  | 0000981 |
|  | 0070412 |
|  | 0001102 |
|  | 0000978 |
|  | 0043425 |
|  | 0008013 |
|  | 0031490 |
|  | 0070410 |
|  | 0005518 |
|  | 0035326 |
|  | 0035259 |
|  | 0031962 |
|  | 0019902 |
|  | 0070878 |
|  | 0046982 |
|  | 0042803 |
|  | 0019901 |
|  | 0000987 |
|  | 0043565 |
|  | 0003712 |
|  | 0008134 |
|  | 0044212 |
|  | 0005160 |
|  | 0030618 |
|  | 0043130 |
|  | 0031625 |
|  | 0008270 |
|  | 0007183 |
|  | 0060395 |
|  | 0042110 |
|  | 0006919 |
|  | 0097296 |
|  | 0032924 |
|  | 0030325 |
|  | 0007050 |
|  | 0045216 |
|  | 0071345 |
|  | 0071560 |
|  | 0048589 |
|  | 0048701 |
|  | 0048617 |
|  | 0009880 |
|  | 0007492 |
|  | 0019049 |
|  | 0097191 |
|  | 0001947 |
|  | 0006955 |
|  | 0002520 |
|  | 0001701 |
|  | 0070306 |
|  | 0001889 |
|  | 0001707 |
|  | 0043066 |
|  | 1903243 |
|  | 0030308 |
|  | 0051481 |
|  | 0045599 |
|  | 0050728 |
|  | 0061767 |
|  | 0045930 |
|  | 0045668 |
|  | 0033689 |
|  | 0042177 |
|  | 0000122 |
|  | 0030512 |
|  | 0061045 |
|  | 0038092 |
|  | 0002076 |
|  | 0048340 |
|  | 0060039 |
|  | 0051091 |
|  | 0010694 |
|  | 0030501 |
|  | 0090263 |
|  | 0030335 |
|  | 0032332 |
|  | 0010718 |
|  | 1901203 |
|  | 0051894 |
|  | 0010628 |
|  | 0032731 |
|  | 0045429 |
|  | 0050927 |
|  | 1902895 |
|  | 0042307 |
|  | 0051496 |
|  | 0045944 |
|  | 0045893 |
|  | 0032916 |
|  | 0031053 |
|  | 0016579 |
|  | 0050821 |
|  | 0051098 |
|  | 0050678 |
|  | 0050776 |
|  | 0016202 |
|  | 0006357 |
|  | 0017015 |
|  | 0032909 |
|  | 0001666 |
|  | 0023019 |
|  | 0001756 |
|  | 0030878 |
|  | 0060290 |
|  | 0007179 |
|  | 0001657 |
|  | 0042060 |
|  | 0071141 |
|  | 0005737 |
|  | 0005829 |
|  | 0071144 |
|  | 0000790 |
|  | 0005637 |
|  | 0005654 |
|  | 0005634 |
|  | 0005886 |
|  | 0043235 |
|  | 0005667 |
|  | 0003677 |
|  | 0000977 |
|  | 0046332 |
|  | 0003682 |
|  | 0003690 |
|  | 0019899 |
|  | 0046872 |
|  | 0007369 |
|  | 0008285 |
|  | 0001649 |
|  | 0006355 |
|  | 0001501 |
|  | 0006366 |
|  | 0032991 |
|  | 0005622 |
|  | 0060070 |
|  | 0000989 |
|  | 0000983 |
|  | 0006351 |
|  | 0035556 |
|  | 0035413 |
|  | 0010467 |
|  | 1901313 |
|  | 0005515 |
|  | 0001836 |
|  | 0000988 |
|  | 0043234 |
|  | 0006810 |
|  | 0001933 |
|  | 0006917 |
|  | 0006367 |
|  | 0008637 |
|  | 0008633 |
|  | 0042993 |
|  | 0010843 |
|  | 0003706 |
|  | 0016566 |
|  | 0016563 |
|  | 0010553 |
|  | 0010552 |
|  | 0045941 |
|  | 0045449 |
|  | 0006350 |
|  | 0015460 |
|  | 0000976 |
|  | 0001223 |

---

|  |  |
| --- | --- |
| KEGG ID | hsa:4088 |
|  | mmu:17127 |
|  | rno:25631 |

---

|  |  |
| --- | --- |
| Organism | Homo sapiens {Organism urn:agi-taxid:9606} |
|  | Mus musculus {Organism urn:agi-taxid:10090} |
|  | Rattus norvegicus {Organism urn:agi-taxid:10116} |
|  | Homo sapiens |
|  | Mus musculus |
|  | Rattus norvegicus |

---

|  |  |
| --- | --- |
| Mouse chromosome position | 9 |

---

|  |  |
| --- | --- |
| OMIM ID | 603109 |
|  | 613795 |

---

|  |  |
| --- | --- |
| Rat chromosome position | 8q24 |

---

|  |  |
| --- | --- |
| Hugo ID | 6769 |
|  | HGNC:6769 |

---

|  |  |
| --- | --- |
| Human chromosome position | 15q22.33 |

---

|  |  |
| --- | --- |
| Swiss-Prot Accession | P84022 |
|  | A0A024R5Z3 |
|  | Q9P0T0 |
|  | P84022.1 |
|  | Q8BUN5 |
|  | Q8BUN5.2 |
|  | P84025 |
|  | P84025.1 |
|  | A8K4B6 |
|  | B7Z4Z5 |
|  | B7Z6M9 |
|  | B7Z9Q2 |
|  | F5H383 |
|  | O09064 |
|  | O09144 |
|  | O14510 |
|  | O35273 |
|  | Q92940 |
|  | Q93002 |
|  | Q9GKR4 |
|  | Q8BX84 |
|  | B1B1A2 |
|  | Q59F45 |
|  | Q68DS8 |
|  | Q05CN3 |
|  | Q3V3E0 |
|  | Q206L0 |
|  | SMAD3\_HUMAN |
|  | SMAD3\_MOUSE |
|  | SMAD3\_RAT |
|  | A8K4B6\_HUMAN |
|  | B7Z4Z5\_HUMAN |
|  | B7Z9Q2\_HUMAN |

---

|  |  |
| --- | --- |
| PIR ID | S71798 |

---

|  |  |
| --- | --- |
| GenBank ID | NC\_000015 |
|  | NM\_005902 |
|  | NP\_005893 |
|  | XM\_011521559 |
|  | XP\_011519861 |
|  | XM\_011521560 |
|  | XP\_011519862 |
|  | NM\_001145102 |
|  | NP\_001138574 |
|  | NM\_001145103 |
|  | NP\_001138575 |
|  | NM\_001145104 |
|  | NP\_001138576 |
|  | NG\_011990 |
|  | AB004930 |
|  | BAA22032 |
|  | AC012568 |
|  | AC087482 |
|  | AH011390 |
|  | AAL68976 |
|  | CH471082 |
|  | EAW77788 |
|  | EAW77789 |
|  | EAW77790 |
|  | EAW77791 |
|  | AA493306 |
|  | AB209616 |
|  | BAD92853 |
|  | AF151027 |
|  | AAF36113 |
|  | AI762628 |
|  | AI991957 |
|  | AK026690 |
|  | AK290881 |
|  | BAF83570 |
|  | AK298139 |
|  | BAH12731 |
|  | AK300614 |
|  | BAH13315 |
|  | AK301348 |
|  | BAH13461 |
|  | AK313812 |
|  | BAG36548 |
|  | AK316017 |
|  | BAH14388 |
|  | AL110265 |
|  | BC000414 |
|  | BC007496 |
|  | BC050743 |
|  | AAH50743 |
|  | BE905995 |
|  | BF058230 |
|  | BG251847 |
|  | BM551682 |
|  | BM722372 |
|  | BQ917888 |
|  | BX506233 |
|  | CR457386 |
|  | CAG33667 |
|  | CR749287 |
|  | CAH18142 |
|  | DA453132 |
|  | DA755047 |
|  | DA977882 |
|  | EU016552 |
|  | EU016553 |
|  | U68019 |
|  | AAB80960 |
|  | U76622 |
|  | AAB18967 |
|  | P84022 |
|  | NC\_000075 |
|  | XM\_006510819 |
|  | XP\_006510882 |
|  | XM\_006510821 |
|  | XP\_006510884 |
|  | NM\_016769 |
|  | NP\_058049 |
|  | AC158515 |
|  | CH466522 |
|  | EDL26045 |
|  | CT010509 |
|  | AB008192 |
|  | BAA76956 |
|  | AF016189 |
|  | AAB81755 |
|  | AK030926 |
|  | AK041743 |
|  | BAE20601 |
|  | AK048626 |
|  | BAC33398 |
|  | AK083158 |
|  | BAC38789 |
|  | AK190092 |
|  | AK190305 |
|  | AK192766 |
|  | AK210600 |
|  | AK219286 |
|  | BC022904 |
|  | AAH22904 |
|  | BC025486 |
|  | BC066850 |
|  | AAH66850 |
|  | Q8BUN5 |
|  | NC\_005107 |
|  | NM\_013095 |
|  | NP\_037227 |
|  | XM\_008766216 |
|  | XP\_008764438 |
|  | AC\_000076 |
|  | AABR07070393 |
|  | AABR07070394 |
|  | AABR07070395 |
|  | AABR07070396 |
|  | AAHX01055130 |
|  | AAHX01055131 |
|  | AAHX01055132 |
|  | AAHX01055133 |
|  | AAHX01055134 |
|  | AAHX01055135 |
|  | AAHX01055136 |
|  | AAHX01055137 |
|  | AAHX01055138 |
|  | AAHX01055139 |
|  | AAHX01055140 |
|  | CH473975 |
|  | EDL95777 |
|  | BC064437 |
|  | AAH64437 |
|  | DQ409172 |
|  | ABD66605 |
|  | FQ232775 |
|  | FQ235081 |
|  | U66479 |
|  | AAC52944 |
|  | P84025 |
|  | AF025297 |
|  | XM\_005254383 |
|  | AF025298 |
|  | AF025295 |
|  | AF025296 |
|  | AF025299 |
|  | XP\_006720569 |
|  | XM\_006243224 |
|  | AMYH02030662 |
|  | AMYH02030663 |
|  | AMYH02030664 |
|  | AF025293 |
|  | AF025294 |
|  | XM\_006510820 |
|  | AC\_000147 |
|  | XP\_006510883 |
|  | AC\_000031 |
|  | XM\_006720506 |
|  | CAM26527 |
|  | CAM26528 |
|  | CAM26529 |
|  | ABBA01038805 |
|  | ABBA01038804 |
|  | CAM26530 |
|  | EU446487 |
|  | NC\_018926 |
|  | AAHY01078810 |
|  | AAHY01078811 |
|  | AAHY01078809 |
|  | AAHY01078807 |
|  | AAHY01078808 |
|  | AAHY01078801 |
|  | AAHY01078802 |
|  | AAHY01078805 |
|  | AAHY01078806 |
|  | AAHY01078803 |
|  | AAHY01078804 |
|  | AABR06055266 |
|  | AABR06055267 |
|  | AABR06055268 |
|  | AABR06055269 |
|  | AABR06055262 |
|  | AABR06055263 |
|  | AABR06055264 |
|  | AABR06055265 |
|  | AABR06055261 |
|  | AF025300 |
|  | XP\_005254440 |
|  | XP\_006243286 |
|  | ABZ92016 |
|  | XM\_008766217 |
|  | XP\_008764439 |
|  | NT\_010194 |
|  | NW\_001838218 |
|  | NW\_925884 |
|  | AC\_000058 |
|  | Q59F45 |
|  | Q68DS8 |
|  | Q9P0T0 |
|  | NT\_039474 |
|  | NW\_001030907 |
|  | Q05CN3 |
|  | Q3V3E0 |
|  | NW\_047799 |
|  | NW\_001084873 |
|  | Q206L0 |

---

|  |  |
| --- | --- |
| Swiss-Prot ID | SMAD3\_HUMAN |
|  | SMAD3\_MOUSE |
|  | SMAD3\_RAT |
|  | B7Z4Z5\_HUMAN |
|  | B1B1A2\_MOUSE |
|  | Q9P0T0\_HUMAN |
|  | B7Z9Q2\_HUMAN |
|  | A8K4B6\_HUMAN |

---

|  |  |
| --- | --- |
| Cell Localization | Cytoplasm |
|  | Nucleus |

---

|  |  |
| --- | --- |
| Ensembl ID | ENSG00000166949 |
|  | ENSP00000437757.2 |
|  | ENST00000540846.6 |
|  | ENSP00000401133.3 |
|  | ENST00000439724.7 |
|  | ENSP00000445348.2 |
|  | ENST00000537194.6 |
|  | ENSP00000332973.4 |
|  | ENST00000327367.9 |
|  | ENSMUSG00000032402 |
|  | ENSMUSP00000034973.3 |
|  | ENSMUST00000034973.9 |
|  | ENSRNOG00000008620 |
|  | ENSRNOP00000037346.3 |
|  | ENSRNOT00000039730.4 |
|  | ENST00000327367.8 |
|  | ENST00000439724 |
|  | ENSRNOT00000039730 |
|  | ENSP00000445348 |
|  | ENSMUSP00000034973 |
|  | ENST00000537194 |
|  | ENSP00000437757 |
|  | ENSMUST00000034973 |
|  | ENSRNOP00000037346 |
|  | ENST00000327367 |
|  | ENSP00000401133 |
|  | ENSP00000332973 |
|  | ENST00000540846 |

---

|  |  |
| --- | --- |
| MGI ID | MGI:1201674 |
|  | 1201674 |

---

|  |  |
| --- | --- |
| RGD ID | 3032 |

---

|  |  |
| --- | --- |
| Unigene ID | Hs.727986 |
|  | Hs.742270 |
|  | Mm.7320 |
|  | Rn.10636 |
|  | Hs.36915 |
|  | Hs.714621 |

---

|  |  |
| --- | --- |
| Homologene ID | 55937 |

---

|  |  |
| --- | --- |
| Shape | O-vertex |

---

|  |  |
| --- | --- |
| IPI ID | IPI00922698 |
|  | IPI00209246 |
|  | IPI00024305 |
|  | IPI00314870 |
|  | IPI00383289 |
|  | IPI00922059 |
|  | IPI00022808 |
|  | IPI00808484 |
|  | IPI00875319 |
|  | IPI00831542 |
|  | IPI00830220 |
|  | IPI00830323 |

---
